# Contemporary Predictors of Major Adverse Cardiovascular Events Following Percutaneous Coronary Intervention: A Nationally Representative US Sample

**DOI:** 10.3390/jcm13102844

**Published:** 2024-05-11

**Authors:** Benjamin D. Horne, Nipun Atreja, John Venditto, Thomas Wilson, Joseph B. Muhlestein, Joshua R. St. Clair, Kirk U. Knowlton, Naeem D. Khan, Narinder Bhalla, Jeffrey L. Anderson

**Affiliations:** 1 Intermountain Medical Center Heart Institute, Salt Lake City, UT 84107, USA; ldbmuhle@gmail.com (J.B.M.); kirk.knowlton@imail.org (K.U.K.); jeffreyl.anderson@imail.org (J.L.A.); 2 Division of Cardiovascular Medicine, Department of Medicine, Stanford University, Stanford, CA 94305, USA; 3 Cardiovascular Institute, Stanford University School of Medicine, Stanford, CA 94305, USA; 4 AstraZeneca Pharmaceuticals LP, Wilmington, DE 19850, USA; nipunatreja@gmail.com (N.A.); john.venditto@astrazeneca.com (J.V.); twilson@trajectory-inc.com (T.W.); joshua.stclair@astrazeneca.com (J.R.S.C.); naeem.khan@astrazeneca.com (N.D.K.); narinder.bhalla@bms.com (N.B.); 5 Cardiology Division, Department of Internal Medicine, University of Utah, Salt Lake City, UT 84132, USA

**Keywords:** PCI, care gaps, CathPCI, clinical outcomes

## Abstract

**Background**: Patient outcomes after percutaneous coronary intervention (PCI) have improved over the last 30 years due to better techniques, therapies, and care processes. This study evaluated contemporary predictors of post-PCI major adverse cardiovascular events (MACE) and summarized risk in a parsimonious risk prediction model. **Methods**: The Cardiovascular Patient-Level Analytical Platform (CLiPPeR) is an observational dataset of baseline variables and longitudinal outcomes from the American College of Cardiology’s CathPCI Registry^®^ and national claims data. Cox regression was used to evaluate 2–6 years of patient follow-up (mean: 2.56 years), ending in December 2017, after index PCI between 2012 and 2015 (N = 1,450,787), to examine clinical and procedural predictors of MACE (first myocardial infarction, stroke, repeat PCI, coronary artery bypass grafting, and mortality). Cox analyses of post-PCI MACE were landmarked 28 days after index PCI. **Results**: Overall, 12.4% (n = 179,849) experienced MACE. All variables predicted MACE, with cardiogenic shock, cardiac arrest, four diseased coronary vessels, and chronic kidney disease having hazard ratios (HRs) ≥ 1.50. Other major predictors of MACE were in-hospital stroke, three-vessel disease, anemia, heart failure, and STEMI presentation. The index revascularization and discharge prescription of aspirin, P2Y_12_ inhibitor, and lipid-lowering medication had HR ≤ 0.67. The primary Cox model had c-statistic c = 0.761 for MACE versus c = 0.701 for the parsimonious model and c = 0.752 for the parsimonious model plus treatment variables. **Conclusions**: In a nationally representative US sample of post-PCI patients, predictors of longitudinal MACE risk were identified, and a parsimonious model efficiently encapsulated them. These findings may aid in assessing care processes to further improve care post-PCI outcomes.

## 1. Introduction

The effective use of clinical data is a critical aspect of precision medical care today for patient risk estimation and the subsequent delivery of best care [1]. Such data utilization includes the application of parameter-specific risk stratifications (especially by giving consideration to risk enhancers) to guide individual diagnostic or therapy decisions [1]. It also includes the employment of risk summation methods to personalize care plans [2,3,4]. The use of patient data for clinical decision-making in practice assumes that the risk information derived from the data represents generalizable risk relationships. Because data-driven improvements to clinical care have contributed over several decades to better outcomes after percutaneous coronary intervention (PCI) and myocardial infarction (MI) [5], those risk relationships may be changing. Other contributors to those changes include improved revascularization techniques, new antiplatelet therapies, and national guidelines [1,6].

While biotechnology advances allow the study today of novel risk factors, few of these are in clinical use. A clinical decision tool for PCI patients, the DAPT Score [2], was developed to guide treatment, but attempts at validation have shown limited utility [3,7]. Further, the use of standard predictor variables to guide care requires knowledge of which parameters continue to predict the risk of major adverse cardiovascular events (MACE). Opportunities exist to better personalize treatment and improve outcomes using contemporary risk information. The objective of this study was to evaluate baseline clinical variables, index angiographic factors, and other hospital encounter variables as contemporary joint predictors of long-term post-PCI MACE in a nationally representative population of patients who underwent PCI in the United States in 2012–2015 and had follow-up ending prior to the pandemic.

## 2. Materials and Methods

### 2.1. Study Objective and Patient Population

The objective of this study was to determine predictors of MACE in patients undergoing PCI using demographic, clinical, procedural, and treatment variables. The primary hypothesis was that among patients undergoing PCI at index hospitalization, the baseline clinical and demographic risk factors, as well as the variables collected during the index PCI and in-hospital findings, results, and other therapies are independent predictors of long-term MACE risk. This was evaluated in a contemporary cohort in which patients were attended to by clinicians who were familiar with possible risk relationships of many standard variables with MACE. The study was conducted according to the principles of Helsinki in the anonymized Cardiovascular Patient-Level Analytical Platform (CLiPPeR) and did not require Institutional Review Board approval due to its analysis of anonymized public-use data, and therefore it was deemed not to constitute human subjects research.

CLiPPeR is an observational dataset of patient characteristics and longitudinal outcomes that were merged from three sources: the American College of Cardiology’s National Cardiovascular Data Registry (NCDR) CathPCI Registry, the NCDR Chest Pain—MI Registry, and the claims data of patients in these registries. It contains information from records of US patients in outpatient pharmacies, private practitioners, and hospitals, attempting to approach the capabilities of datasets available in countries with nationalized healthcare. This study included patients from the CathPCI Registry, which was begun in the 1990s, and today contains data on millions of patients [6,8,9]. The CLiPPeR dataset was constructed to empower the evaluation of clinically important hypotheses in real-world data from a large, nationally representative US population of patients treated according to standard care processes. CLiPPeR resulted from the collaborative efforts of Symphony Health Solutions and AstraZeneca.

All study patients underwent index PCI as the primary entry criterion. Index PCIs occurred between 1 January 2012 and 31 December 2015, and they had to be the first PCI for each patient recorded in the study period. One additional year of PCI data existed (for 2011), which was used to evaluate whether a prior PCI had occurred, and anyone who had a PCI during 2011 was excluded from study analyses. Prior revascularization, either via PCI or coronary artery bypass grafting (CABG), was assessed further by evaluating codes indicating that revascularization had occurred at some remote time prior to 2011. Because of the lack of availability of data prior to 2010, the pertinent dates and other information were not available, and patients were not excluded based on this limited information. At least two years of follow-up time had to exist for patients to be included in the study, and up to almost 6 years of follow-up were possible. Patients had to have one or more discharge medication prescriptions to be included.

### 2.2. Study MACE Outcome

All follow-up for longitudinal events was completed on 31 December 2017, which was the study end date and censor date for study subjects who did not have MACE during follow-up. Study analyses were landmarked at 28 days after the index PCI to ensure that the three non-fatal endpoints in the MACE outcome (i.e., MI, stroke, and repeat revascularization, which were defined based on International Classification of Disease version 9 [ICD-9] codes: 410.x for MI, 433.x1, 434.x1, or 436 for stroke; V45.81 or V45.82 for revascularization) were long-term events. Due to the landmarking, all patients included in this study survived to at least 29 days after PCI, and only events occurring after 28 days post-PCI were counted as outcomes. MACE outcomes after 28 days were also required to have an inpatient ICD-9 discharge diagnosis code that was a primary diagnosis for the post-PCI encounter. MI, stroke, and revascularization events occurring on or prior to 28 days were considered a technical failure of the index therapy or staged PCI procedures and were excluded from the study. Mortality was the fourth endpoint included as an event in the MACE outcome composite. All events in the study were considered long-term outcomes occurring after the 28-day landmark. All occurrences and procedures that were experienced during the baseline hospitalization, including in-hospital “events” such as stroke or major bleeding were considered independent risk factors that could affect the long-term risk profile and were not outcome events per se in this study.

Among subjects who did not experience a MACE endpoint, the determination that they were lost to follow-up was based on the date of the last claim available from any facility, prescription source, or contact note with a health professional as recorded in the CLiPPeR dataset, or it was the study end date if the last claim occurred thereafter. Patients with zero claims of any kind during the study follow-up period who did not die were excluded from the study as being lost to follow-up prior to the end of the initial 28-day post-PCI study exclusion period.

***Study Predictor Variables***. Study variables included demographics and clinical factors, including the following: subject age, sex, race, obesity (body mass index ≥ 30 kg/m^2^), anemia (hemoglobin < 12.0 g/dL for females or <13.5 g/dL for males), smoking history, hypertension history, dyslipidemia history, previous diagnosis of diabetes, prior MI, prior stroke, prior PCI, prior CABG, previous diagnosis of heart failure (HF), history of peripheral arterial disease (PAD), chronic kidney disease (CKD: estimated glomerular filtration rate < 60 mL/min/1.73 m^2^), current treatment with dialysis, or history of chronic lung disease (CLD). The history of a diagnosis was defined based on text information in the datasets for diagnosis of the condition at index or prior diagnosis, and thus medical history variables were not based on ICD-9 codes. Further, interventional variables and in-hospital findings were also examined: clinical indication for PCI (ST-elevation MI [STEMI], non-ST-elevation MI [NSTEMI], or other), creatine kinase myocardial band (CK-MB) (troponins were not routinely available in this study’s time frame), presentation with cardiogenic shock, presentation with cardiac arrest, maximum coronary stenosis, in-hospital peripheral thromboembolic event, in-hospital stroke, in-hospital major bleeding, and in-hospital blood transfusion. Treatment variables included discharge prescriptions of aspirin, P2Y_12_ inhibitor/thienopyridine, statin or other lipid-lowering medication, or angiotensin-converting enzyme (ACE) inhibitor or angiotensin receptor blocker (ARB), stent placement at index PCI, and CABG during index admission (including hybrid revascularization and failed PCI, which unfortunately could not be separated in these analyses). The maximum stenosis was categorized as the presence of moderate coronary narrowing (50% to <70% stenosis in the left anterior descending, left circumflex, or right coronary artery) as the most severe lesion, or the number of severely diseased vessels (severe lesions were ≥70% stenosis in the left anterior descending, left circumflex, or right coronary artery, or ≥50% stenosis in the left main). Four diseased vessels included the left main. Variables with missing data included anemia, CK-MB, and discharge medications, and missing elements were designated as not documented to allow all subjects to be included in the statistical modeling and, thus, to obtain optimal estimates of MACE risk; all other variables had full data.

### 2.3. Other Statistical Considerations

Evaluations of associations between study variables and MACE were performed initially by the chi-square test. Cox regression was used to model multivariable associations with MACE using time-to-event data in survival analyses. Stepwise regression analysis was utilized to initially assess the strength of association for each variable and whether it contributed to the model. Reduced variable models were used to evaluate the association of dyslipidemia and dialysis with MACE. Final Cox modeling added variables using a forced entry approach. Using a standard to a conservative rule of thumb of 15 to 20 MACE outcomes per variable, the 179,849 MACE outcomes in the study permitted between 9000 and 12,000 independent variables to be entered simultaneously into Cox regression (if they had been available).

Further analyses were performed to create a parsimonious risk model that included a subset of the more important independent variables based on the Cox regression beta-coefficients of the risk predictors, with scalar weightings of 1, 2, 3, or 4 assigned to risk factors with hazard ratios (HRs) = 1.25–1.49, 1.50–1.74, 1.75–1.99, and ≥2.00, respectively. The scalar weights for each risk factor were then added up for each patient, resulting in a range of 0–19 across the population. A parsimonious model is one in which fewer than all of the available or even all of the statistically significant independent variables are entered into the model while a similar level of risk prediction ability by the parsimonious model is realized compared to the full model. These Cox models excluded variables with HRs of 1.01–1.24 or 0.81–0.99 as having lower clinical significance that did not appreciably affect the summarized risk of the model, and all variables with HR ≤ 0.80 were excluded because they were treatment variables that were collected at discharge after the major decisions regarding the in-hospital treatment approach and the patient’s plan for post-discharge care had been made (although factors such as NSTEMI and one-vessel disease were included in the multivariable Cox model used to assign scalar weightings because they were dummy variables related to STEMI and two-, three-, and four-vessel disease; in addition, the referent group would change if they were excluded, but they were assigned weightings of zero). Harrell’s concordance (c)-statistic was calculated for the resultant risk model and patients with similar risk were grouped based on considerations of the HR compared to the lowest risk group and taking into account the sample size that would result for the category. Sensitivity analyses evaluated subsets defined by antiplatelet medication prescriptions and bleeding status at index hospitalization. Statistical analyses used SAS, and nominal statistical significance was defined as *p* ≤ 0.05, although all variables were statistically significant in the modeling at each Cox analysis stage.

## 3. Results

Of N = 1,450,787 post-PCI patients included in the study, 12.4% (n = 179,849) experienced MACE during long-term follow-up. Subjects were followed for a mean (±standard deviation) of 2.56 ± 1.40 years among those who did not experience an event, while those who experienced MACE had the first event 1.37 ± 1.28 years after index PCI. Subjects averaged 66 years of age, and 31.9% were female. Other baseline demographics and clinical characteristics are provided in Table 1, listing overall data and the results stratified by MACE. Variables collected during the hospitalization for the index PCI included MI characteristics, angiographic findings, PCI results, short-term in-hospital outcomes, and discharge treatments. These data are provided in Table 2. Notably, all variables were statistically significant predictors of the MACE outcome in univariable analysis, demonstrating the limitation of the *p*-value as a measure of biological significance in very large populations.

**Figure 1:**
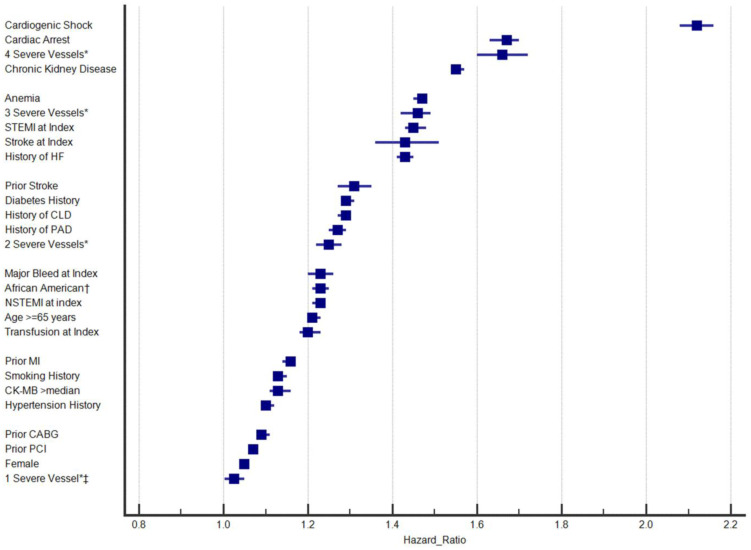
Forest plot for contemporary predictors of higher risk of MACE that shows hazard ratios (HRs) and 95% confidence intervals (CIs) for each risk predictor; * compared to moderate coronary disease (i.e., a severely diseased vessel was defined as a coronary narrowing of ≥70% stenosis in the left anterior descending, left circumflex, or right coronary artery or ≥50% stenosis in the left main coronary artery, while moderate coronary disease constituted maximum stenosis of at least 50% but less than 70% in all of the major branches and <50% in the left main); † compared to Caucasian; ‡ all comparisons of statistical significance had *p* < 0.001 except for the comparison (*p* = 0.026) of 1 severely diseased coronary vessel versus moderate coronary disease. Abbreviations: CABG: coronary artery bypass graft, CK-MB: creatine kinase myocardial band, CLD: chronic lung disease, HF: heart failure, MI: myocardial infarction, NSTEMI: non-ST-elevation myocardial infarction, PAD: peripheral arterial disease, PCI: percutaneous coronary intervention, STEMI: ST-elevation myocardial infarction.

**Figure 2:**
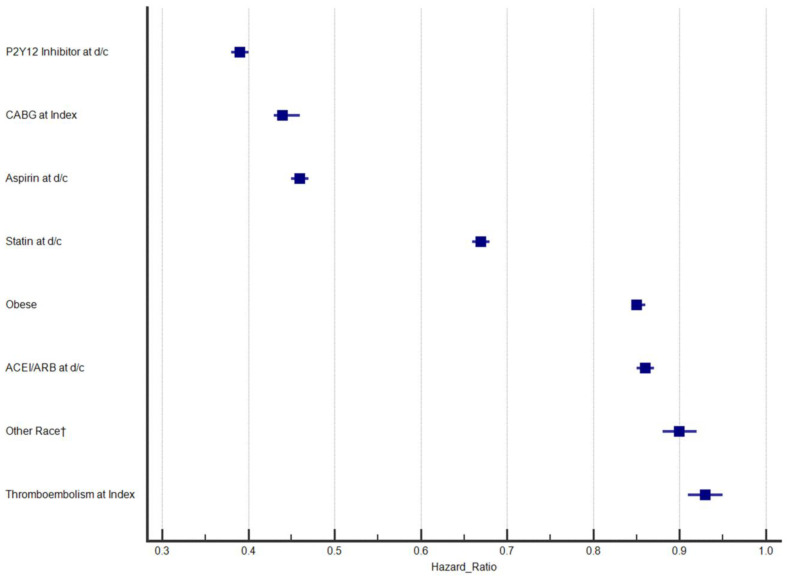
Forest plot of the hazard ratios (HRs) and 95% confidence intervals (CIs) for variables predictive of lower risk of MACE in this contemporary observational cohort; † compared to Caucasians. Abbreviations: ACEI: angiotensin-converting enzyme inhibitor, ARB: angiotensin receptor blocker, CABG: coronary artery bypass graft, d/c: discharge.

MACE outcomes during long-term follow-up (>28 days after the index PCI) for the 12.4% who had events were 1.31% (n = 19,039) repeat PCI, 0.036% (n = 521) CABG, 0.0065% (n = 94) both repeat PCI and CABG, 3.05% (44,202) MI (incident or recurrent), 1.58% (n = 22,922) stroke, 0.0094% (n = 136) both MI and stroke, and 6.41% (n = 92,935) mortality. The association results for variables that predicted MACE in the final multivariable Cox regression model for predictors of greater risk of MACE are shown in Figure 1, with Figure 2 providing results for predictors of lower MACE risk. The strongest predictors of MACE were cardiogenic shock at index hospitalization and, in a distant second, cardiac arrest. Closely behind was the category regarding four significantly diseased coronary vessels (including the left main). HRs and 95% confidence intervals (CIs) for each predictor are reported in Table 3. CKD was fourth to round out the list of factors with HRs ≥ 1.50. Four variables had HR ≤ 0.67, including discharge prescription of a P2Y_12_ inhibitor, discharge prescription of aspirin, discharge prescription of a statin/lipid-lowering medication, and CABG at index hospitalization. Twenty variables had HR > 0.67 to <0.90 or HR > 1.10 to <1.50 (Figure 1). Clinically minimal effects (i.e., HR ≥ 0.90 and ≤1.10) were found for seven variables. The only variable that did not predict a difference in MACE was index stent placement (*p* = 0.49).

Dyslipidemia history was present among 72.2% of patients and had an HR = 0.88 (95% confidence interval [CI] = 0.87, 0.89) in multivariable modeling. Receipt of a discharge prescription for a statin or other lipid-modifying therapy had an HR = 0.69 (CI = 0.68, 0.70) for MACE when dyslipidemia was entered in the Cox modeling and MACE when dyslipidemia was entered in the Cox modeling and an HR = 0.67 (CI = 0.66, 0.68) when dyslipidemia was excluded. Because of this and the frequent clinical recording of a diagnosis of dyslipidemia as justification for lipid-lowering/statin therapy, dyslipidemia was excluded from the final Cox model. Similarly, dialysis had an HR = 1.41 (CI = 1.39, 1.44) in multivariable Cox modeling, suggesting that this variable indicated those subjects with severe CKD. In Cox modeling, the association of CKD with MACE was HR = 1.46 (CI = 1.44, 1.47) when dialysis was included in the model and CKD had an HR = 1.55 (CI = 1.54, 1.57) without dialysis in the model. Because dialysis was a wholly enclosed subset of CKD and constituted only 2.9% of patients, and the association of CKD appeared to encapsulate the dialysis risk, dialysis was eliminated from the final Cox model. For variables with missing data, the no-documentation category’s HRs were tracked with those of patients recorded as having the comorbidity or characteristic (Supplementary Table S1). 

The full multivariable Cox model (Table 3) had a c-statistic of c = 0.761 for MACE. In comparison, the parsimonious risk model with no treatment variables had c = 0.701 and the parsimonious model including treatment variables (i.e., CABG at index and prescription of aspirin, P2Y_12_ inhibitor, and statin at discharge) had c = 0.752 (just 0.009 lower than the full model). Table 4 contains the association results from Cox regression including the four treatment variables. When those treatment variables were added to the model, only the associations of the higher risk scalar model categories (i.e., categories ≥ 9) with MACE were substantially influenced by adjustment for the four treatment variables, with the HR for the category of 9–11 reduced from HR = 9.76 to 7.88, and for 12–19, the HR reduced from HR = 25.17 to 12.55.

The evaluation of treatment-based considerations provided further insights into risk prediction, with MACE occurring in 10.2%, 12.9%, 12.7%, and 60.8% of patients receiving aspirin and a P2Y_12_ inhibitor, aspirin alone, a P2Y_12_ inhibitor alone, and neither aspirin nor a P2Y_12_ inhibitor, respectively. In sensitivity analyses excluding patients not receiving aspirin or a P2Y_12_ inhibitor or excluding patients with major bleeding at index hospitalization, the association of the parsimonious model with MACE remained strong (Supplementary Table S2).

Importantly, while major bleeding during the index admission was associated with a higher risk of MACE, clinical decisions regarding which patients received prescriptions for single or dual-antiplatelet therapy compared to no antiplatelet agent were associated with MACE risk regardless of bleeding status, with patients not receiving aspirin or a P2Y_12_ inhibitor having substantially elevated MACE risk (Supplementary Table S3). The parsimonious model strongly stratified MACE risk regardless of bleeding status or antiplatelet prescription (Supplementary Table S3). Further, major bleeding at index was substantially associated with discharge antiplatelet prescription (17.9% of subjects who bled versus 3.8% of those who did not bleed received no antiplatelet prescription, although this meant that 82.1% of patients with bleeding did receive an antiplatelet prescription [including 72.9% dual-antiplatelet therapy, 5.8% aspirin alone, and 3.4% only a P2Y_12_ inhibitor]), but subjects with and without bleeding were similarly stratified by the parsimonious model, especially in those not receiving antiplatelet prescriptions (Supplementary Table S4).

## 4. Discussion

In a fully pre-pandemic analysis of almost one and a half million US patients who underwent PCI during 2012–2015 and were followed for 2–6 years, major risk predictors associated with MACE during long-term follow-up were cardiogenic shock, cardiac arrest, severe stenosis in four coronary vessels (including the left main), and CKD. Other risk predictors were anemia, three-vessel disease, STEMI, in-hospital stroke, and previous diagnosis of HF. Predictors of lower risk included discharge prescriptions of guideline-directed medical therapies. To characterize clinically meaningful differences, a parsimonious model was developed from factors that had HR ≥ 1.25 and were shown to have a similar ability to discriminate MACE risk as the risk model that included all risk predictors.

While the proliferation of possible predictors of MACE has occurred in the past decade, including proteomic, metabolomic, genomic, and microbiomic factors, common demographics, clinical factors, procedural data, and in-hospital outcomes have historically been reliable indicators of patient risks and needs for care. The risk predictors of long-term outcomes after PCI were investigated early in the experience with percutaneous intervention to aid in stratifying risk and improving care [10]. Additional work over the decades has evaluated risk prediction in patients undergoing PCI, including changing standard processes of care and guideline-directed medical therapy and the associated short- and long-term outcomes [11,12]. In the present study, the evaluated predictive abilities foster an understanding of recent healthcare practices and areas where improvement in care quality may be achieved. For example, multivessel disease was found to have elevated risks associated with four-vessel disease (HR = 1.66), three-vessel disease (HR = 1.46), and two-vessel disease (HR = 1.25). This angiographic risk factor may be utilized to target continued care process improvement efforts to reduce major adverse patient outcomes. Other independent risk factors, including age, sex, history of hypertension, smoking history, and prior MI, were associated with a higher risk of MACE herein and could be similarly used to guide care improvement.

Beyond these factors, the predictors of post-PCI mortality in the DAPT Study were recently found to include STEMI and NSTEMI, while univariate predictors of ischemic events (not including mortality or revascularization) included demographics, cardiac risk factors, and procedural and lesion characteristics [11]. In that analysis, all participants from the trial, including those randomized to the placebo and treatment arms, were included, medications prescribed at index including P2Y_12_ inhibitors were not evaluated as predictors, and multivariable analyses were not performed for long-term prediction of ischemic events [11]. Further, a recent evaluation of the CathPCI Registry has updated the multivariable predictors of in-hospital mortality after PCI but not long-term predictors of MACE outcomes [12]. This highlights the need for the present study to update predictive models for long-term MACE risk after PCI. The data from the CathPCI Registry provide a powerful mechanism for doing so.

The CathPCI Registry has changed the conversation for understanding patients undergoing PCI and their post-PCI risks [6]. While many of the risk predictors that were previously evaluated are used in the present analysis, this study updates those associations and provides contemporary estimates of relative hazards within a rank order. It also adds additional predictors to the modeling, including new discharge prescriptions (e.g., P2Y_12_ inhibitors that were unavailable in the 1980s and 1990s), and provides better resolution on estimates of effect with a substantially larger population from centers across the US. Importantly, the present study reveals how guideline-directed medical therapy is associated with lower MACE risk. Notably, given that all patients in this study underwent PCI, the prescription of P2Y_12_ inhibitors may be an indicator of clinical selection for therapy and reveal that those not receiving P2Y_12_ inhibitors may have had contraindications such as a major bleed prior to discharge.

Insights from this study’s results can be used to further improve our understanding of the needs of PCI patients, leading to modified care and reduced MACE incidence in future patients. This is particularly the case in the use of these data for clinical risk stratification to identify gaps in care or understanding. Specifically, the risk associations may be used to guide the development of clinical risk scores to estimate long-term MACE risk, similar to the use of the DAPT Score for short-term risk [2]. In this context, such a score would be used in broad applications (i.e., not just for a single medication) [1]; that is, the breadth of data on multiple risk predictors herein could assist in the precise targeting of which patient should receive priority attention and to whom should greater clinician time be devoted for personalizing evaluation and treatment [3,4]. Further, the derivation of separate risk scores for subpopulations (e.g., using distinct variable weightings for males and females) or the evaluation of the risk scores in subgroups would be key.

The parsimonious model reported here was associated with MACE risk regardless of index bleeding status. Further, it stratified MACE risk in patients receiving or not receiving discharge prescriptions for antiplatelet agents. This model revealed that MACE risk was profoundly influenced by clinical selection for antiplatelets and that selection was only minimally influenced by actual major bleeding. While some patients not receiving antiplatelets might have been referred to hospice, these data suggest that decisions to withhold antiplatelet therapy may be influenced by perceived bleeding risk rather than actual bleeding. While this requires further investigation, modeling may have identified a critical care gap where fear of bleeding leads to a lack of chemoprophylaxis in 3–4% of patients and, thus, to substantial MACE risk. This is supported by the finding that in 76% of de-escalations from a more potent P2Y_12_ inhibitor to clopidogrel after MI for which the rationale was not documented, 15% were due to cost, and only 6% were due to observed bleeding [13].

Finally, females had a 5% higher MACE risk, and African Americans had a 23% higher risk. While these were not among the most substantial risk associations, these continued distinctions in outcomes should not be ignored in clinical care. These risk relationships may in part reflect sociocultural factors but also likely reveal health system considerations regarding how symptoms, health status, and perception of risk are considered and managed. It may be that enhanced or more thoughtful application of current tools for risk modification could improve outcomes without excessive effort from clinicians.

### Strengths and Limitations

This study is limited by the non-randomized observational nature of the dataset. The most important limitations of the study include the fact that the follow-up period is relatively short, the results apply to outcomes in the first few years after PCI, and the clinical relevance of a prediction model for a heterogeneous PCI population for individual patient decision-making is difficult to assess. Further, the analyses may be limited by failure to include all possible predictors of MACE (e.g., atrial fibrillation, valve disease, etc.) and by potential residual confounding. In part, issues may exist due to the clinical assignment of treatments, as well as missing data in discharge medications where patients with MACE were more likely to be missing such information. Further, some angiographic variables (e.g., lesion complexity, stent characteristics, and completeness of revascularization), electrocardiographic data, and laboratory testing results (such as lipid profiles and the complete blood count) were not available here, but were identified previously by other analyses as predictors of MACE [11,14]. In part, this inhibited the calculation of other risk models such as the TIMI Risk Score for comparison purposes. CABG at index likely encapsulated patients with failed PCI and those who had hybrid revascularization, which, in the univariate analysis, did not predict MACE differences but, in multivariable analysis, predicted a lower risk of MACE. This may have arisen from adjustment for factors enriched in patients with failed PCI and due to having no variable for the receipt of revascularization. The results for CABG at index should be interpreted with caution. Also, historical diagnoses such as heart failure are risk predictors in these analyses, while heart failure is also predicted by other modeled risk factors (e.g., anemia and hypertension); thus, although multivariable analyses should remove most confounding, some residual complexity may remain. This may be pertinent to cardiogenic shock, a risk factor that imbues patients with such higher risk that previous analyses considered shock patients separately from the general PCI population.

The modeling of the left main disease has limitations since it feeds two major coronary arteries; thus, other methods of modeling may have given different findings. Also, the low hazard ratios for prescriptions of P2Y_12_ inhibitors, aspirin, and statins may be more profound than the expected benefits, with results arising in part from selection by physicians for those who receive prescriptions based on underlying conditions other than coronary disease. Unfortunately, data regarding medication adherence were not available for this study. Since the long-term effectiveness of treatment depends on the actual use of medications, and the average patient is known to not fully adhere to prescribed therapies, future studies should gather adherence data to optimally describe the association of pharmaceutical variables with MACE outcomes. Further, data on prescriptions of anticoagulants were not available and, thus, some of the protection against MACE and some risk of bleeding may have arisen from sources other than the antiplatelets evaluated here. The inclusion of patients from across the US may have introduced unstandardized treatment differences between institutions with different geographies, prehospital services (e.g., ambulances or air transport), and in-hospital treatment practices. However, such distinctions should have resulted in limited variation in outcomes since the acute failure of care in the first 28 days after PCI was excluded. Similar considerations exist for pre-hospital time from symptom onset and time since risk factor development varies between patients.

The focus of this project was on the composite MACE endpoint, and analyses of each component of MACE were not performed; thus, the important clinical differences between the component outcomes and the varied frequency of each outcome may provide risk prediction results that relate to a heterogeneous endpoint rather than the severity and clinical features of each outcome. For example, approximately half of the study’s MACE outcomes were deaths from all causes. Prior studies reported that more than half of deaths in cardiac patients were due to non-cardiac causes [15]. Any non-cardiac deaths may have impacted which variables predicted MACE; thus, caution is urged with respect to causal inferences from this study’s findings. In contrast, this study’s results have importance for real-world practice where some PCI patients die of non-cardiac causes. Notably, while most staged PCI procedures were excluded as long-term PCI events due to the 28-day landmark, a small proportion of events after 28 days may still have been staged PCI.

The strengths of this study were that it included more than one million patients, and the population was drawn from a broad collection of centers from across the US. Also, survival analyses included a wealth of variables previously connected to the risk of MACE.

## 5. Conclusions

In a nationally representative, pre-pandemic US sample of post-PCI patients constituting the largest-ever long-term post-PCI outcomes study, index events (i.e., cardiogenic shock, cardiac arrest, and in-hospital stroke), coronary anatomy (four- and three-vessel disease), non-coronary comorbid conditions (CKD, anemia, and HF), and presentation with STEMI were the most powerful predictors of longitudinal MACE risk. History of stroke and diabetes also predicted elevated risk, with tight confidence intervals (e.g., CI = 1.28, 1.31 for diabetes). Discharge medications and CABG at index (likely primarily representing hybrid PCI) were the most powerful predictors of a lower risk of MACE. In the subgroup not receiving antiplatelets, MACE risk was profoundly elevated, but only a small proportion had evidence of major bleeding; thus, further investigation is needed regarding the withholding of antiplatelet prescriptions and the appropriate balance of MACE incidence and bleeding risk. This study also suggests that future development of clinical risk scores in the post-PCI population may use fewer than all possible risk predictors to adequately describe the risk of MACE, and thus, it further empowers the creation of risk scores that are calculated and delivered to the point of care by the electronic health record. Further work is needed to derive and externally validate feasible clinical risk scores in post-PCI patients to improve care processes and ameliorate the long-term risk of MACE. For conditions that have stronger risk associations with MACE such as shock, cardiac arrest, anemia, or 3–4-vessel disease, a focused risk prediction model in just those with the condition may provide enhanced clinical usefulness to identify the characteristics associated with MACE—especially those that are modifiable factors to which treatments or prevention may be most effectively targeted in the patient subsets.

## Figures and Tables

**Table 1:** Baseline demographics and clinical characteristics of the study population. Subjects with and without MACE were followed for a mean ± SD of 1.37 ± 1.28 years and 2.56 ± 1.40 years, respectively, after index PCI.

Characteristic	Overall	No MACE	MACE	*p*-Value
Sample size	1,450,787	1,270,938	179,849	-----
Age ≥ 65 years	795,160 (54.8%)	680,284 (53.5%)	114,876 (63.9%)	<0.001
Sex (female)	463,250 (31.9%)	399,254 (31.4%)	63,996 (35.6%)	<0.001
Race				
African American	125,275 (8.6%)	104,821 (8.3%)	20,454 (11.4%)	<0.001
Caucasian	1,2590,135 (86.8%)	1,107,032 (87.1%)	152,103 (84.6%)	
Other	66,377 (4.6%)	59,085 (4.7%)	7292 (4.1%)	
Obese (BMI ≥ 30 kg/m^2^)	613,664 (42.3%)	544,023 (42.8%)	69,641 (38.7%)	<0.001
Smoking History	396,393 (27.3%)	346,273 (27.3%)	50,120 (27.9%)	<0.001
Hypertension History	1,171,056 (80.7%)	1,017,840 (80.1%)	153,216 (85.2%)	<0.001
Dyslipidemia History	1,054,990 (72.7%)	926,314 (72.9%)	128,676 (71.6%)	<0.001
Diabetes History	531,597 (36.6%)	447,206 (35.2%)	84,391 (46.9%)	<0.001
Cardiogenic Shock	36,940 (2.6%)	19,742 (1.6%)	17,198 (9.6%)	<0.001
Cardiac Arrest	37,081 (2.6%)	23,360 (1.8%)	13,721 (7.6%)	<0.001
Prior MI	352,101 (24.3%)	295,590 (23.3%)	56,511 (31.4%)	<0.001
Prior PCI	446,250 (30.8%)	383,431 (30.2%)	62,819 (34.9%)	<0.001
Prior CABG	211,850 (14.6%)	174,483 (13.7%)	37,367 (20.8%)	<0.001
Prior Stroke	16,396 (1.1%)	10,930 (0.9%)	5466 (3.0%)	<0.001
History of HF	168,161 (11.6%)	130,902 (10.3%)	37,259 (20.7%)	<0.001
History of CLD	208,637 (14.4%)	171,011 (13.5%)	37,626 (20.9%)	<0.001
History of PAD	164,614 (11.4%)	131,859 (10.4%)	32,755 (18.2%)	<0.001
CKD (eGFR < 60 *)	442,521 (30.5%)	352,676 (27.8%)	89,845 (50.0%)	<0.001
Dialysis	44,829 (3.1%)	29,799 (2.3%)	15,030 (8.4%)	<0.001
Anemia †				
No	949,348 (65.4%)	857,912 (67.5%)	91,436 (50.8%)	<0.001
Yes	444,847 (30.7%)	362,822 (28.6%)	82,025 (45.6%)	
Not Documented	56,592 (3.9%)	50,204 (4.0%)	6388 (3.6%)	

* mL/min/1.73 m^2^; † hemoglobin < 12.0 g/dL for females or <13.5 g/dL for males. Abbreviations: BMI: body mass index, CABG: coronary artery bypass graft, CKD: chronic kidney disease, CLD: chronic lung disease, eGFR: estimated glomerular filtration rate, HF: heart failure, MI: myocardial infarction, PAD: peripheral arterial disease, PCI: percutaneous coronary intervention, SD: standard deviation.

**Table 2:** Encounter-related factors at the index PCI describing the hospitalization course across the presentation, catheterization procedure, and discharge of the study population.

Characteristic	Overall	No MACE	MACE	*p*-Value
Sample size	1,450,787	1,270,938	179,849	-----
Indication for PCI				
STEMI	283,306 (19.5%)	237,964 (18.7%)	45,342 (25.2%)	<0.001
NSTEMI	805,766 (55.5%)	706,648 (55.6%)	99,118 (55.1%)	
Other	361,715 (24.9%)	326,326 (25.68%)	35,389 (19.7%)	
CK-MB (>median)				
No	202,213 (13.9%)	175,217 (13.8%)	26,996 (15.0%)	<0.001
Yes	199,425 (13.8%)	166,618 (13.1%)	32,807 (18.2%)	
Not Documented	1,049,149 (72.3%)	929,103 (73.1%)	120,046 (66.8%)	
Extent of Coronary Artery Disease *				
Moderate Stenosis	345,076 (23.8%)	294,364 (85.3%)	50,712 (14.7%)	<0.001
1 Severe Vessel	613,754 (42.3%)	556,901 (43.8%)	56,853 (31.6%)	
2 Severe Vessels	322,343 (22.2%)	280,815 (22.1%)	41,528 (23.1%)	
3 Severe Vessels	146,178 (10.1%)	120,624 (9.5%)	25,554 (14.2%)	
4 Severe Vessels	23,436 (1.6%)	18,234 (1.4%)	5202 (2.9%)	
Stent(s) at Index	1,432,859 (98.8%)	1,257,447 (98.9%)	175,412 (97.5%)	<0.001
CABG at Index	18,609 (1.3%)	16,005 (1.3%)	2604 (1.5%)	<0.001
Index Thromboembolism	133,711 (9.2%)	110,410 (8.7%)	23,301 (13.0%)	<0.001
Major Bleed at Index	29,653 (2.0%)	21,671 (1.7%)	7982 (4.4%)	<0.001
Transfusion at Index	36,379 (2.5%)	24,108 (1.9%)	12,271 (6.8%)	<0.001
Stroke at Index	4677 (0.3%)	2750 (0.2%)	1927 (1.1%)	<0.001
Aspirin Prescribed at Discharge				
No	77,323 (5.3%)	47,148 (3.7%)	30,175 (16.8%)	<0.001
Yes	1,354,138 (93.3%)	1,215,356 (95.6%)	138,782 (77.2%)	
No Documentation	19,326 (1.3%)	8434 (0.7%)	10,892 (6.1%)	
P2Y_12_ Inhibitor/Thienopyridine Prescribed at Discharge				
No	71,938 (5.0%)	42,709 (3.4%)	29,229 (16.3%)	<0.001
Yes	1,354,216 (93.3%)	1,215,338 (95.6%)	138,878 (77.2%)	
No Documentation	24,633 (1.7%)	12,891 (1.0%)	11,742 (6.5%)	
Statin/Lipid-Lowering Medication Prescribed at Discharge				
No	144,419 (10.0%)	106,417 (8.4%)	38,002 (21.1%)	<0.001
Yes	1,070,547 (73.8%)	970,128 (76.3%)	100,419 (55.8%)	
No Documentation	235,821 (16.3%)	194,393 (15.3%)	41,428 (23.0%)	
ACE Inhibitor or ARB Prescribed at Discharge				
No	472,857 (32.6%)	405,547 (31.9%)	67,310 (37.4%)	<0.001
Yes	897,618 (61.9%)	804,459 (63.3%)	93,159 (51.8%)	
No Documentation	80,312 (5.5%)	60,932 (4.8%)	19,380 (10.8%)	

* A severely diseased vessel was defined as a coronary narrowing of ≥70% stenosis in the left anterior descending, left circumflex, or right coronary artery or ≥50% stenosis in the left main coronary artery, while moderate coronary disease constituted a maximum stenosis of at least 50% but less than 70% in all of the major branches and <50% in the left main. Abbreviations: ACE: angiotensin-converting enzyme, ARB: angiotensin receptor blocker, CABG: coronary artery bypass graft, CK-MB: creatine kinase myocardial band, MI: myocardial infarction, NSTEMI: non-ST-elevation myocardial infarction, PCI: percutaneous coronary intervention, STEMI: ST-elevation myocardial infarction.

**Table 3:** Multivariable Cox regression results for variables that were associated with incident MACE during longitudinal follow-up (landmarked at 28 days post-PCI).

Study Variable	HR (95% CI)	Study Variable	HR (95% CI)
Variables Associated with a Higher Risk of MACE			
Cardiogenic Shock *	2.12 (2.08, 2.16)		
Cardiac Arrest *	1.67 (1.63, 1.70)		
4 Severe Vessels * †	1.66 (1.60, 1.72)		
CKD *	1.55 (1.54, 1.57)		
Anemia*	1.47 (1.45, 1.48)		
3 Severe Vessels * †	1.46 (1.42, 1.49)		
STEMI at Index *	1.45 (1.43, 1.48)		
Stroke at Index *	1.43 (1.36, 1.51)		
History of HF *	1.43 (1.41, 1.45)		
Prior Stroke *	1.31 (1.27, 1.35)		
Diabetes History *	1.29 (1.28, 1.31)		
History of CLD *	1.29 (1.27, 1.30)		
History of PAD *	1.27 (1.25, 1.29)		
2 Severe Vessels * †	1.25 (1.22, 1.28)		
Major Bleed at Index	1.23 (1.20, 1.26)		
African American ‡	1.23 (1.21, 1.25)		
NSTEMI at Index	1.23 (1.21, 1.24)		
Age ≥ 65 years	1.21 (1.20, 1.23)		
Transfusion at Index	1.20 (1.18, 1.23)		
Prior MI	1.16 (1.14, 1.17)		
Smoking History	1.13 (1.12, 1.15)		
CK-MB (>median)	1.13 (1.11, 1.16)		
Hypertension History	1.10 (1.09, 1.12)		
Prior CABG	1.09 (1.08, 1.11)		
Prior PCI	1.07 (1.06, 1.08)		
Female	1.05 (1.04, 1.06)		
1 Severe Vessel † §	1.025 (1.003, 1.05)		
Variables Associated with a Lower Risk of MACE			
P2Y_12_ Inhibitor at d/c	0.39 (0.38, 0.40)	Obese	0.85 (0.846, 0.86)
CABG at Index	0.44 (0.43, 0.46)	ACEI/ARB at d/c	0.86 (0.85, 0.87)
Aspirin at d/c	0.46 (0.45, 0.47)	Other Race ‡	0.90 (0.88, 0.92)
Statin at d/c	0.67 (0.66, 0.68)	TE at Index	0.93 (0.91, 0.95)

* Included as a component of the parsimonious risk model; † compared to moderate coronary disease (i.e., a severely diseased vessel was defined as a coronary narrowing of ≥70% stenosis in the left anterior descending, left circumflex, or right coronary artery or ≥50% stenosis in the left main coronary artery, while moderate coronary disease constituted maximum stenosis of at least 50% but less than 70% in all of the major branches and <50% in the left main); ‡ compared to Caucasians; § all comparisons of statistical significance had *p* < 0.001 except for the comparison (*p* = 0.026) of 1 severely diseased coronary vessel versus moderate coronary disease. Abbreviations: ACEI: angiotensin-converting enzyme inhibitor, ARB: angiotensin receptor blocker, CABG: coronary artery bypass graft, CI: confidence interval, CK-MB: creatine kinase myocardial band, CKD: chronic kidney disease, CLD: chronic lung disease, d/c: discharge, HF: heart failure, HR: hazard ratio, MI: myocardial infarction, NSTEMI: non-ST-elevation myocardial infarction, PAD: peripheral arterial disease, PCI: percutaneous coronary intervention, STEMI: ST-elevation myocardial infarction, TE: thromboembolism.

**Table 4:** Association of the parsimonious model with MACE in Cox regression.

		Univariable HR (95% CI)		Multivariable HR (95% CI)	
Predictor	MACE *		*p*-Value		*p*-Value
Parsimonious Model Scalar Risk Categories					
0–1	5.5%	1.0 (referent)	-----	1.0 (referent)	-----
2	7.6%	1.37 (1.35, 1.40)	<0.001	1.42 (1.39, 1.45)	<0.001
3	9.6%	1.76 (1.73, 1.80)	<0.001	1.75 (1.72, 1.78)	<0.001
4	12.3%	2.28 (2.23, 2.32)	<0.001	2.28 (2.24, 2.32)	<0.001
5–6	17.0%	3.33 (3.27, 3.38)	<0.001	3.25 (3.19, 3.30)	<0.001
7–8	24.6%	5.33 (5.24, 5.43)	<0.001	4.96 (4.88, 5.05)	<0.001
9–11	36.8%	9.76 (9.57, 9.94)	<0.001	7.88 (7.73, 8.03)	<0.001
12–19	59.4%	25.17 (24.44, 25.91)	<0.001	12.55 (12.18, 12.94)	<0.001
P2Y_12_ Inhibitor at d/c	NR	-----	-----	0.40 (0.39, 0.40)	<0.001
Aspirin at d/c	NR	-----	-----	0.43 (0.42, 0.43)	<0.001
CABG at Index	NR	-----	-----	0.45 (0.44, 0.47)	<0.001
Statin at d/c	NR	-----	-----	0.53 (0.53, 0.54)	<0.001

* Cumulative MACE incidence is provided over the full follow-up time of the study in the following sample sizes for the indicated categories of the parsimonious model (based on the scalar risk values): 0–1: n = 385,138; 2: n = 234,105; 3: n = 235,749; 4: n = 173,981; 5–6: n = 234,756; 7–8: n = 118,244; 9–11: n = 59,070; and 12–19: n = 9744. Abbreviations: CABG: coronary artery bypass graft, CI: confidence interval, d/c: discharge, HR: hazard ratio, MACE: major adverse cardiovascular event, NR: not reported.

## Data Availability

The authors do not have permission to share the data used in this study. The data are available by contacting their respective owners, including the American College of Cardiology (https://cvquality.acc.org/NCDR-Home, accessed on 10 May 2024) and sources of national claims data.

## Supplementary Materials

The following supporting information can be downloaded at: https://www.mdpi.com/article/10.3390/jcm13102844/s1, Table S1. Associations of not having documentation for those variables that were missing data. Table S2. Sensitivity analysis results. Table S3. Association of discharge prescription status for aspirin and a P2Y12 inhibitor (P2Y12-I) with major adverse cardiovascular events (MACE) and of the parsimonious model with MACE in discharge prescription subgroups. Table S4. Across each category of the parsimonious risk model, (A) the risk of major bleeding at index and (B) the distribution of patients with major bleeding at index among subgroups defined by discharge prescriptions for antiplatelet medications.
