# Supplementary material for: Contemporary Predictors of Major Adverse Cardiovascular Events Following Percutaneous Coronary Intervention: A Nationally Representative US Sample
Source: J Clin Med. 2024 May 11;13(10):2844. doi: 10.3390/jcm13102844 (PMC11121929; doi:10.3390/jcm13102844)
Supplement: Supplementary file 1 [file jcm-13-02844-s001.zip › jcm-2994049-supplementary.pdf]

Supplemental Materials for the Study:

Contemporary Predictors of Major Adverse Cardiovascular Events following Percutaneous Coronary Intervention: Nationally Representative US Sample

Supplemental Table S1. Associations of not having documentation for those variables that were missing data. Cox survival analyses compared the “No Documentation” category to the variable’s referent in which the referent was not having the condition (e.g., no anemia) or not receiving the prescription.

Variable	Patients for Whom the Status was Not Documented	Not Documented vs. Referent HR (95% CI)	p-value
Anemia	4%	1.19 (1.16, 1.22)	p<0.001
CK-MB*	72%	1.10 (1.09, 1.12)	p<0.001
Aspirin at discharge	1%	1.48 (1.43, 1.53)	p<0.001
$P2Y_{12}$ inhibitor at d/c	2%	0.81 (0.79, 0.84)	p<0.001
ACEI/ARB at d/c	5%	1.02 (0.997, 1.05)	p=0.08
Statin/lipid-lowering medication at d/c	16%	1.36 (1.32, 1.40)	p<0.001

*Referent category was CK-MB ≤median.

Abbreviations: ACEI: angiotensin-converting enzyme inhibitor, ARB: angiotensin receptor blocker, CI: confidence interval, CK-MB: creatine kinase myocardial band, d/c: discharge, HR: hazard ratio, MI: myocardial infarction, NSTEMI: non-ST elevation myocardial infarction, PAD: peripheral arterial disease, PCI: percutaneous coronary intervention, STEMI: ST-elevation myocardial infarction.

Supplemental Table S2. Sensitivity analysis results. Associations of the parsimonious model with MACE in: A) Cox regression excluding 59,652 patients who did not receive aspirin or a $P2Y_{12}$ inhibitor ($P2Y_{12}$-I) at discharge from the index hospital encounter (c=0.682), and B) Cox regression excluding 29,653 patients who experienced major bleeding during the index hospitalization prior to the receipt of any discharge prescriptions (c=0.745) (compare Table 3).

	**A) Prescribed Aspirin and/or $P2Y_{12}$ Inhibitor**		**B) Free of Major Bleeding at Index**	
**Predictor**	**MACE***	**HR (95% CI), p-value**	**MACE†**	**HR (95% CI), p-value**
Parsimonious Model Scalar Risk Categories				
0-1	5.3%	1.0 (referent), -----	5.5%	1.0 (referent), -----
2	7.3%	1.42 (1.39, 1.45), <0.001	7.6%	1.42 (1.39, 1.45), <0.001
3	8.6%	1.69 (1.65, 1.72), <0.001	9.5%	1.74 (1.70, 1.77), <0.001
4	11.0%	2.21 (2.16, 2.25), <0.001	12.1%	2.26 (2.22, 2.31), <0.001
5-6	14.7%	3.10 (3.04, 3.15), <0.001	16.7%	3.22 (3.17, 3.28), <0.001
7-8	20.2%	4.69 (4.60, 4.78), <0.001	24.1%	4.96 (4.87, 5.05), <0.001
9-11	25.7%	6.79 (6.64, 6.95), <0.001	36.1%	7.97 (7.81, 8.13), <0.001
12-19	27.1%	8.40 (7.94, 8.88), <0.001	59.1%	13.27 (12.9, 13.7), <0.001
$P2Y_{12}$-I at d/c	NR	0.87 (0.85, 0.90), <0.001	NR	0.41 (0.40, 0.41), <0.001
Aspirin at d/c	NR	0.89 (0.87, 0.90), <0.001	NR	0.44 (0.43, 0.45), <0.001
CABG at Index	NR	0.60 (0.57, 0.64), <0.001	NR	0.44 (0.42, 0.46), <0.001
Statin at d/c	NR	0.59 (0.58, 0.60), <0.001	NR	0.53 (0.53, 0.54), <0.001

*In analyses excluding patients who did not receive aspirin or a $P2Y_{12}$ inhibitor, sample sizes for the parsimonious model scalar risk categories were, for scores of 0-1: n=379,431; 2: n=230,013; 3: n=229,236; 4: n=168,355; 5-6: n=223,329; 7-8: n=108,221; 9-11: n=47,762; and 12-19: n=4,788; †In analyses excluding patients who experienced major bleeding during the index hospitalization, sample sizes for the parsimonious model scalar risk categories were, for scores of 0-1: n=381,125; 2: n=231,350; 3: n=231,526; 4: n=170,579; 5-6: n=228,491; 7-8: n=113,840; 9-11: n=55,531; and 12-19: n=8,692.

Abbreviations: CABG: coronary artery bypass graft, CI: confidence interval, d/c: discharge, HR: hazard ratio, MACE: major adverse cardiovascular events, NR: not reported, $P2Y_{12}$-I: $P2Y_{12}$ Inhibitor.

Supplemental Table S3. Association of discharge prescription status for aspirin and a $P2Y_{12}$ inhibitor ($P2Y_{12}$-I) with major adverse cardiovascular events (MACE) and of the parsimonious model with MACE in discharge prescription subgroups. Cumulative incidence of MACE over the full study follow-up time are provided.

	**Free of Major Bleeding at Index**		**Had Major Bleeding at Index**	
**Variable**	**Sample**	**MACE**	**Sample**	**MACE**
A) Medications and MACE				
Aspirin and $P2Y_{12}$-I	1,295,592	130,896 (10.1%)	21,627	3,195 (14.8%)
Aspirin Alone	35,214	4,411 (12.5%)	1,705	280 (16.4%)
$P2Y_{12}$-I Only	35,977	4,593 (12.8%)	1,020	194 (19.0%)
No Aspirin, No $P2Y_{12}$-I	54,351	31,967 (58.8%)	5,301	4,313 (81.4%)
B) Parsimonious Model and MACE				
Scalar Risk Categories in Patients with Aspirin, a $P2Y_{12}$ Inhibitor, or Both				
0-1	367,348	19,186 (5.2%)	3,611	235 (6.5%)
2	222,438	16,028 (7.2%)	2,428	234 (9.6%)
3	219,472	18,834 (8.6%)	3,444	375 (10.9%)
4	160,921	17,607 (10.9%)	2,695	369 (13.7%)
5-6	211,703	30,955 (14.6%)	4,788	860 (18.0%)
7-8	101,665	20,439 (20.1%)	3,145	684 (21.8%)
9-11	43,909	11,321 (25.8%)	2,112	517 (24.5%)
12-19	4,113	1,119 (27.2%)	424	115 (27.1%)
Scalar Risk Categories in Patients Not Prescribed Aspirin or a $P2Y_{12}$ Inhibitor				
0-1	13,777	1,798 (13.1%)	402	117 (29.1%)
2	8,912	1,515 (17.0%)	327	111 (33.9%)
3	12,054	3,063 (25.4%)	779	391 (50.2%)
4	9,658	3,013 (31.2%)	707	395 (55.9%)
5-6	16,788	7,206 (42.9%)	1,477	952 (64.5%)
7-8	12,175	7,031 (57.8%)	1,259	927 (73.6%)
9-11	11,622	8,730 (75.1%)	1,427	1,166 (81.7%)
12-19	4,579	4,022 (87.8%)	628	534 (85.0%)

Abbreviations: MACE: major adverse cardiovascular events, $P2Y_{12}$-I: $P2Y_{12}$ Inhibitor.

Supplemental Table S4. Across each category of the parsimonious risk model, A) the risk of major bleeding at index and B) the distribution of patients with major bleeding at index among subgroups defined by discharge prescriptions for antiplatelet medications. Although the statistical association of major bleeding at index hospitalization with the parsimonious risk model were significant ($p<0.001$ in both medication subgroups), clinically-relevant differences were minimal in the distribution of patients with and without major bleeding across scalar risk categories in those not prescribed aspirin or a $P2Y_{12}$ inhibitor.

	**Parsimonious Model Scalar Risk Categories**							
**Medication Subgroup**	**0-1**	**2**	**3**	**4**	**5-6**	**7-8**	**9-11**	**12-19**
A) Major Bleeding in Patients Receiving a Prescription for:								
**Aspirin, a $P2Y_{12}$ Inhibitor, or Both**	1.01%	1.93%	2.87%	3.01%	3.69%	4.32%	6.72%	12.7%
**No Aspirin or $P2Y_{12}$ Inhibitor**	3.14%	3.91%	7.35%	8.35%	9.57%	10.5%	11.3%	11.8%
B) Distribution of Patients in Medication Subgroups based on Bleeding Status								
**Patients Receiving Prescription for Aspirin, a $P2Y_{12}$ Inhibitor, or Both**								
Free of Major Bleeding at Index	27.5%	16.6%	16.5%	12.1%	16.0%	7.7%	3.3%	0.3%
Had Major Bleeding at Index	15.7%	10.7%	15.4%	12.0%	21.2%	13.8%	9.3%	1.9%
**Patients Not Prescribed Aspirin or a $P2Y_{12}$ Inhibitor**								
Free of Major Bleeding at Index	10.2%	7.2%	11.1%	9.5%	19.0%	16.5%	18.5%	8.0%
Had Major Bleeding at Index	3.4%	3.0%	9.0%	8.9%	20.6%	19.9%	24.2%	11.0%
